# Supplementary material for: Algorithm for Schroth-Curve-Type Classification of Adolescent Idiopathic Scoliosis: An Intra- and Inter-Rater Reliability Study
Source: Children (Basel). 2023 Mar 8;10(3):523. doi: 10.3390/children10030523 (PMC10047876; doi:10.3390/children10030523)
Supplement: Supplementary file 1 [file children-10-00523-s001.zip › Supplementary Material S2.pdf]

Supplementary Material S2

Operational definitions for Schroth Classification Algorithm

NOTE: The algorithm has been developed in a way so that it detects the presence or absence of a certain category.

Main (major) curve:

The main curve is a structural scoliosis curvature, which is more pronounced and less flexible compared to other existent scoliotic curves in the spine of a patient with scoliosis [1]. According to the SOSORT terminology, “major scoliosis curvature is the curve with the largest Cobb measurement on upright long cassette coronal x-ray of the spine”[2]. According to the Schroth method, the main curve influences the body static the most. [1]

Apex:

The apex, or apical vertebra, is the most laterally displaced and rotated vertebra relative to the vertical axis of the body that passes through the patient’s sacrum, and compared to the other vertebrae included in the scoliosis curve. [2]

Thoracic curve:

A thoracic scoliosis curvature is any spinal curvature in which the apex of the curve is between the second (T2) and eleventh thoracic (T11) vertebrae [1,2].

Lumbar curve:

A lumbar scoliosis curve is a spinal curvature whose apex is between the first (L1) and fourth lumbar (L4) vertebrae (also known as lumbar scoliosis) [1,2].

<u>Thoracolumbar curve:</u>

A thoracolumbar curve is any spinal curvature that has its apex at the twelfth thoracic (T12) or first lumbar (L1) vertebra [2].

<u>Concave:</u>

Curving inward or curved like the inner surface of a sphere. In scoliosis, the concave side is the inner side of the curve [2].

<u>Convex:</u>

A surface or boundary that curves or bulges outward, as the exterior of a sphere. In scoliosis, the convex side is the outer side of the curve [2].

<u>Rib hump:</u>

A rib hump is observed on the patient's upper back side of the trunk at the level of the thoracic curve. It is located only on one side of the upper back, and is the most prominent area compared to the opposite side at the same level. A rib hump is located on the thoracic convex side of the body [1,3].

- Steps in assessment of the rib hump:
    - A patient assumes her/his habitual posture, while the therapist observes the upper back, searching for asymmetry between left and right side of the patient's body.

- In the presence of a rib hump, one side of the upper back will be more prominent and the shoulder blade will be protracted on that side.
- The Adam's forward bend test in standing should be performed on a patient, and asymmetry should be searched for between the left and right sides. In this test, the rib hump is the most visible as it protrudes the most while trunk is bent and palms are firmly pressed against each other.
- If a clear decision cannot be made, use the scoliometer to assess the largest vertebral rotation in the upper back. If the number reads 7 or more, a rib hump is present. Positive numbers on the scoliometer refer to right asymmetry, and negative to left asymmetry.

<u>Lumbar prominence:</u>

A lumbar prominence is located in the lower back at the level of the lumbar scoliosis curve. It is the most prominent (protruding) area of the lower back observed unilaterally. It is positioned on the thoracic concave side of the body [1,3].

- Steps in assessment of the lumbar prominence:
  - A patient assumes her/his habitual posture, while the therapist observes the lower back, searching for asymmetry between left and right sides of the patient's body.
  - In the presence of a lumbar prominence, one side of the lower back is more prominent and the waist becomes asymmetric. Also, the lumbar convex side appears as if swollen, while the lumbar concave side is indented.
  - The Adam's forward bend test in standing should be performed on a patient, and asymmetry should be searched for between the left and right lower back sides.

- If a clear decision cannot be made, use the scoliometer to assess the largest vertebral rotation in the lower back. If the number reads 7 or more, a lumbar prominence is present. Positive numbers on the scoliometer refer to a right asymmetry, and negative to a left asymmetry.

<u>Prominent hip:</u>

A prominent hip represents the compensation for either thoracic or lumbar major curves [1]. When the pelvis laterally deviates in the frontal plane, the prominent hip will occur. Because the pelvis is laterally shifted, the hip becomes adducted (on the side to which the pelvis is shifted) and the iliac crest on the same side becomes raised [1]. A prominent hip, according to the Schroth classification system, only appears in the 3cp and 4cp scoliosis curve patterns [1,3].

- Steps in assessment of the prominent hip:
    - A patient assumes her/his habitual posture, while the therapist observes the hips and pelvis, searching for asymmetry between the left and right sides of the patient's body.
    - The therapist should look for triangles bounded by the arms hanging loosely at the patient' s sides and the corresponding side of the pelvis. If the pelvis is closer to the arm on the thoracic concave side, the prominent hip will be present on the same side. If the pelvis deviates more to the arm on the thoracic convex side, the prominent pelvis will be present on that side.
    - In case the asymmetry is not so obvious, a therapist should use a plumb line suspended from the spinous process of C7 to the gluteal cleft to observe lateral hip deviation. The prominent hip will be observed on the side to which the hip deviates, with reference to the plumb line.

- In the presence of a prominent hip, weight bearing is asymmetrically distributed. A Schroth therapist could use two scales of the same brand and ask a patient to step on each with one leg. If the scales read differently, and more weight is distributed on the thoracic convex side, the prominent hip is on the opposite side. If more weight occurs on the thoracic concave side, the prominent hip is observed on the thoracic convex side.

<u>Pelvis balanced:</u>

Pelvic balance is closely related to the prominent hip. In the absence of a prominent hip, the pelvis is referred to as balanced.

<u>Pelvis unbalanced:</u>

Pelvic balance is closely related to the prominent hip. In the presence of a prominent hip, the pelvis is referred to as unbalanced.

<u>Pelvis coupled with the lumbar spine:</u>

Pelvic displacement can be coupled with the deviation of the lumbar spine, due to scoliosis. The pelvis is considered coupled with the lumbar spine if it deviates to the same side as the lumbar spine.

- Steps in the assessment of the coupled mechanism of the pelvis and lumbar spine:
    - If the pelvis is judged as unbalanced, there are two possible outcomes:

1. When the prominent hip is observed on the thoracic concave side, the pelvis will be coupled with the lumbar spine, as it will deviate in the same direction as the lumbar convexity.
2. When the prominent hip is observed on the thoracic convex side, the pelvis will be uncoupled with the lumbar spine, as it will deviate in the opposite direction of the lumbar convexity.

<u>Pelvis uncoupled with the lumbar spine:</u>

Pelvic displacement can be uncoupled with the deviation of the lumbar spine, due to scoliosis. The pelvis is considered uncoupled with the lumbar spine if it is deviated to the opposite side of the lumbar spine. If uncoupled, the pelvis can deviate towards the midline, or further away, in the opposite direction of the lumbar spine deviation.

- Steps in the assessment of the uncoupled mechanism of the pelvis and lumbar spine:
  - If the pelvis is deemed as balanced in the physical assessment, then it will always be uncoupled with the lumbar spine, as it leans towards the midline, and away from the lumbar convex scoliosis curve.

Supplementary material 2 - References

1. Hennes A. *Schroth-Method*. Bad Sobernheim: Asklepios Katharina Schroth Klinik; 2011.
2. Grivas TB, de Mauroy JC, Negrini S, et al. Terminology - glossary including acronyms and quotations in use for the conservative spinal deformities treatment: 8th SOSORT consensus paper. 2010;5:23. doi:10.1186/1748-7161-5-23.
3. Lehnert-Schroth C. *Three-Dimensional Treatment for Scoliosis*. 7 ed. Palo Alto, California, USA: The Martindale Press; 2007.
