# Supplementary material for: Algorithm for Schroth-Curve-Type Classification of Adolescent Idiopathic Scoliosis: An Intra- and Inter-Rater Reliability Study
Source: Children (Basel). 2023 Mar 8;10(3):523. doi: 10.3390/children10030523 (PMC10047876; doi:10.3390/children10030523)

Supplementary Material S1

Snapshots of the video assessment.

Supplementary Figure S1: Presentation of a 3c curve type as determined by the experienced rater.

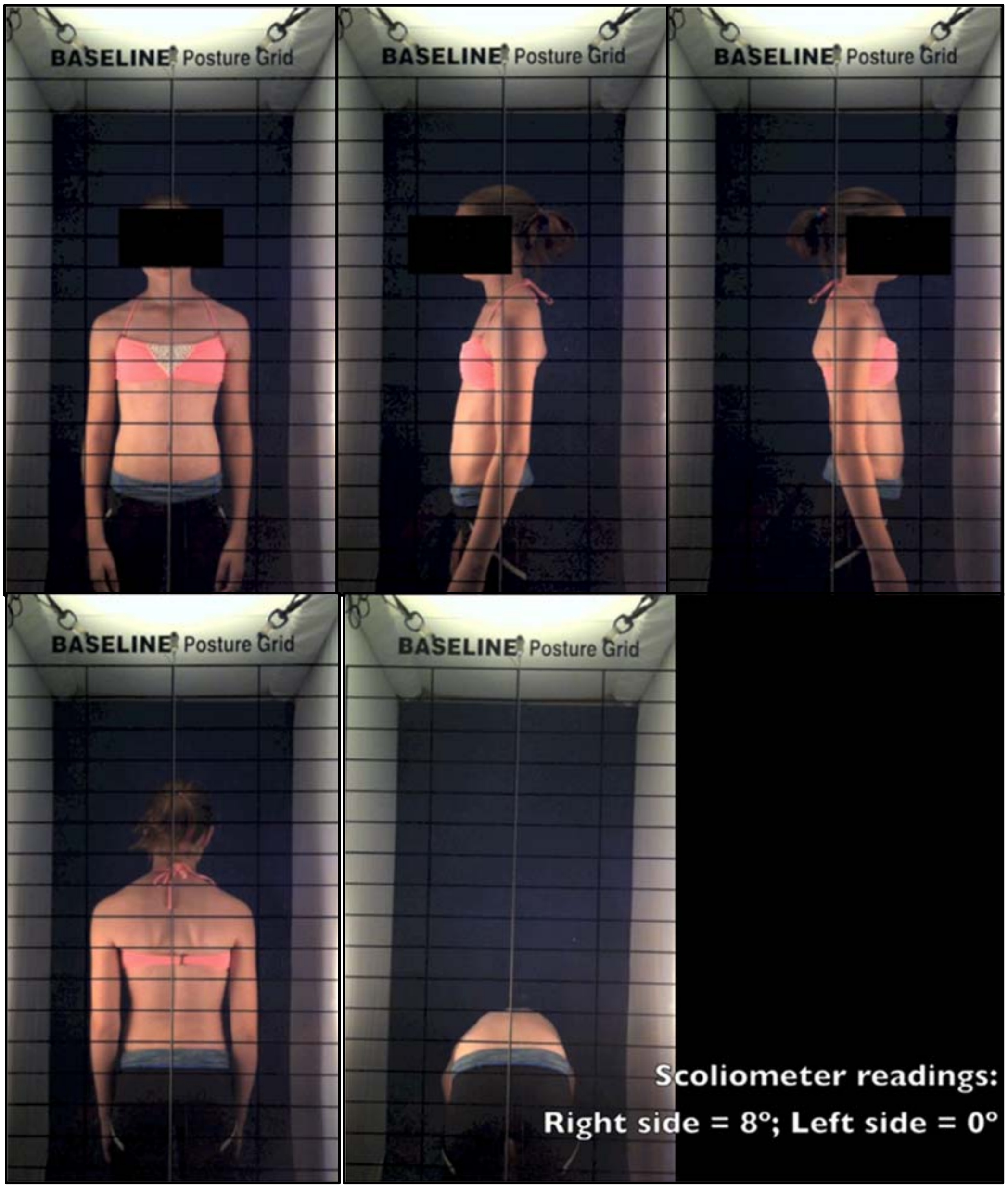

Supplementary Figure S2: Presentation of a 4c curve type as determined by the experienced rater.

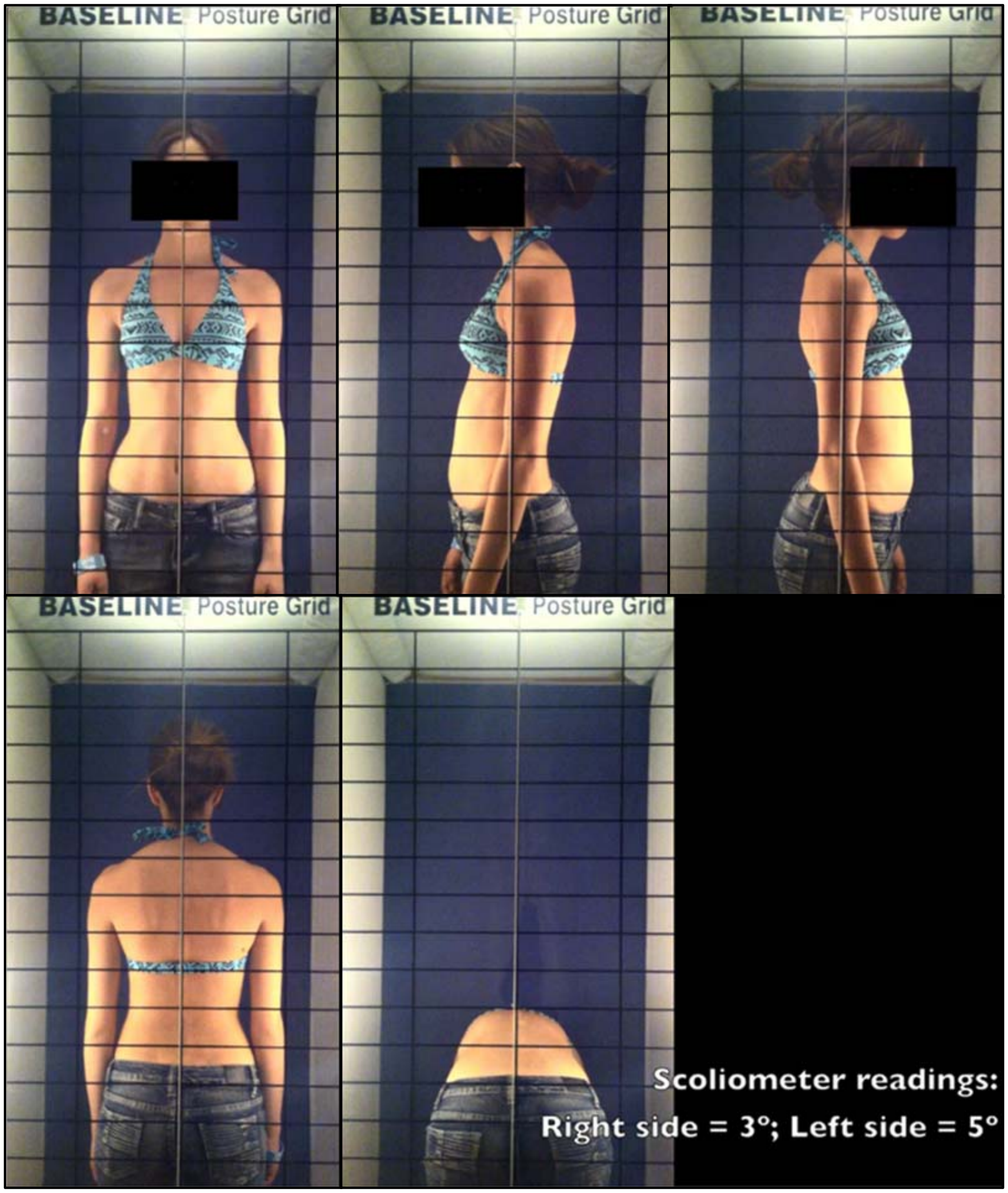

Supplementary Figure S3: Presentation of a 4cp curve type as determined by the experienced rater.

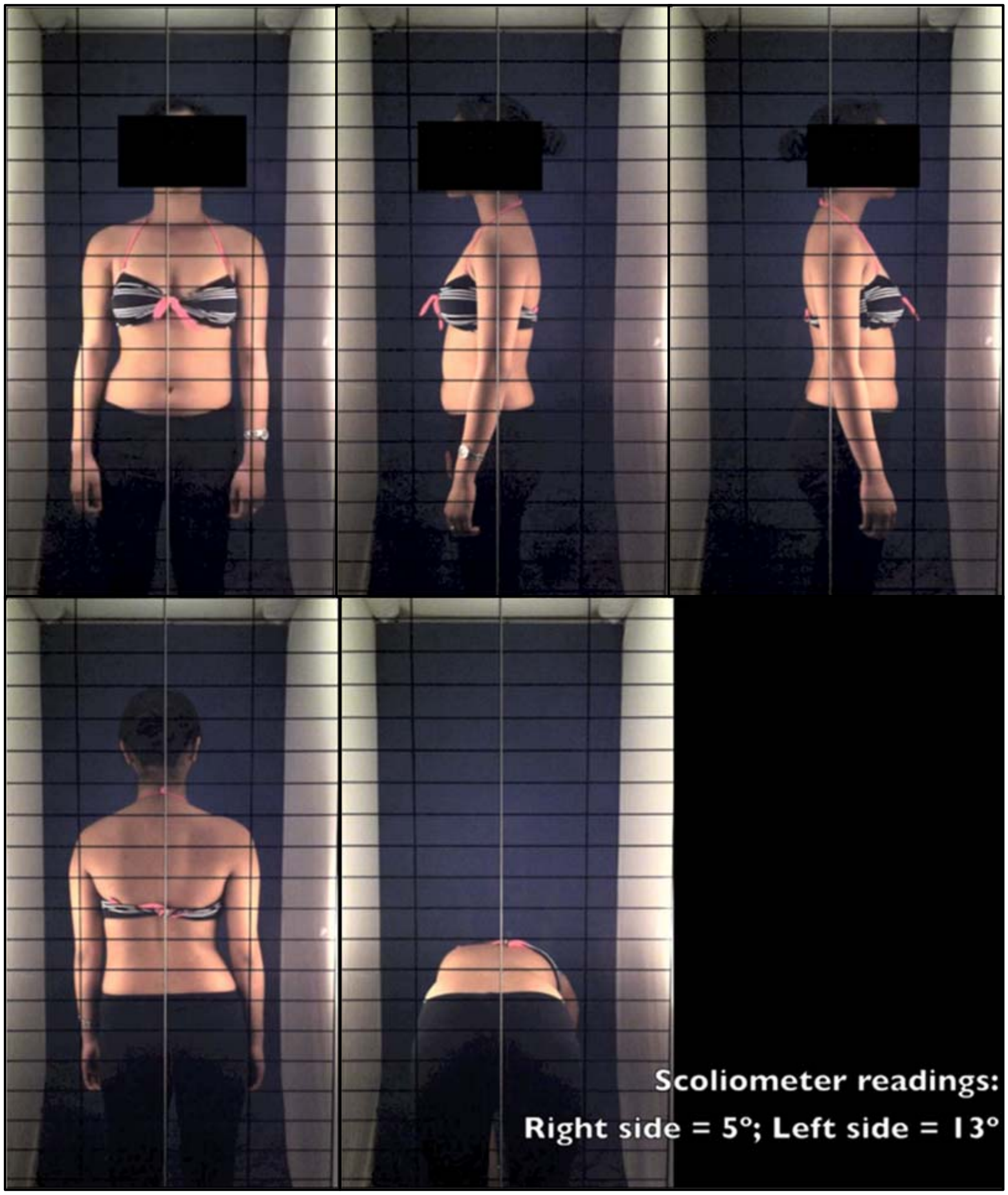

Supplement: Supplementary file 1 [file children-10-00523-s001.zip › Supplementary Material S1.pdf]
